# Does Chemotherapy for Gynecological Malignancies during Pregnancy Cause Fetal Growth Restriction?

**DOI:** 10.1155/2017/7543421

**Published:** 2017-05-24

**Authors:** Nabil Abdalla, Magdalena Bizoń, Robert Piórkowski, Paweł Stanirowski, Krzysztof Cendrowski, Włodzimierz Sawicki

**Affiliations:** Chair and Department of Obstetrics, Gynecology and Oncology, Medical University of Warsaw, Kondratowicza Street 8, 03-242 Warsaw, Poland

## Abstract

Cancer and pregnancy rarely coincide. Gynecological cancers are among the most common malignancies to occur during pregnancy, and chemotherapy with or without surgery is the primary treatment option. The main concern of administering chemotherapy during pregnancy is congenital malformation, although it can be avoided by delaying treatment until after organogenesis. The dose, frequency, choice of chemotherapeutic agents, time of treatment commencement, and method of administration can be adjusted to obtain the best maternal treatment outcomes while simultaneously minimizing fetal toxicity. Use of chemotherapy after the first trimester, while seemingly safe, can cause fetal growth restriction. However, the exact effect of chemotherapy on such fetal growth restriction has not been fully established; information is scarce owing to the rarity of malignancy occurring during pregnancy, the lack of uniform treatment protocols, different terminologies for defining certain fetal growth abnormalities, the influence of mothers' preferred options, and ethical issues. Herein, we present up-to-date findings from the literature regarding the impact of chemotherapy on fetal growth.

## 1. Introduction

Malignancies rarely coincide with pregnancies; only 1 in 1000 pregnancies occur concurrently with cancer. The most common malignancies diagnosed during pregnancy are breast cancer, cervical cancer, Hodgkin's lymphoma, and leukemia [1].

Small for gestational age (SGA) refers to an infant born with a birth weight below the 10th centile. The centile is adjusted to the maternal characteristics, gestational age, and the sex of the fetus. “Severe SGA” is defined as an estimated fetal weight or abdominal circumference below the 3rd centile. SGA is not synonymous with fetal growth restriction (FGR); the latter refers to a pathological restriction of the genetic growth potential and may manifest as evidence of fetal compromise such as abnormal Doppler findings or reduced amniotic fluid volume. Low birth weight refers to an infant with a birth weight less than the absolute value of 2500 g. Definitions for fetal growth abnormalities may vary in the literature, and the term FGR may even be used inappropriately [2]. Frequently, abnormally restricted growth has been referred to as intrauterine growth restriction (IUGR) in the literature and was previously known as intrauterine growth retardation [3]. Large for gestational age refers to infants with weights above the 90th centile for the gestational age and is not to be confused with macrosomia, which is defined as a fetal weight above an absolute value. Different absolute values have been used in the literature, ranging from 4000 g to 4500 g at birth [4].

The main drawback of administering chemotherapy during pregnancy is the development of congenital abnormalities. However, chemotherapy after the first trimester is not associated with increasing the rates of birth defects above the 3% rate found in the general population [5]. The exact incidence of fetal growth abnormalities caused by chemotherapy has not been established. IUGR is a possible complication of chemotherapy according to the largest medical registry in the literature, in which Cardonick and Iacobucci analyzed 376 fetuses exposed to chemotherapy in utero and showed the following complications: IUGR (7%), spontaneous rupture of membranes or preterm labor (5%), fetal death (5%), and neonatal death (1%). In terms of congenital malformations, 9 of the 11 cases of congenital malformations were attributed to chemotherapy in the first trimester. Most fetal and neonatal deaths were related to maternal hematological malignancies, and 2 deaths were related to idarubicin treatment for breast cancer [6]. In 2010, Van Calsteren et al. presented the results of their analysis of pregnancies complicated by malignancies in 3 European countries. The authors showed that, considering the cytotoxic treatment (chemotherapy and/or radiotherapy), SGA babies were observed significantly more often in 16 of 66 pregnancies (24.2%, *p* = 0.001) versus in 10 of 109 pregnancies (9.2%) without cytotoxic treatment where the association was not significant [7]. Results of the analysis of an American registry of perinatal outcomes describing 152 pregnant women managed with chemotherapy were presented in 2010, where the mean gestational age at delivery for fetuses exposed to chemotherapy was 35.8 ± 2.8 weeks and the mean birth weight was 2647 ± 713 g. Six children (3.8%) were born with a congenital anomaly. One case each of intrauterine fetal death and neonatal death occurred (0.7% of fetuses each). In 12 cases (7.7%), the neonate had SGA. The authors compared their results to those of 67 pregnant women who did not receive chemotherapy during pregnancy and concluded that congenital abnormalities, IUGR, and preterm deliveries were not increased among pregnancies managed with chemotherapy after the first trimester compared to the rates in the general population. However, there was a significant statistical difference in birth weight between the groups, although they may not be clinically consequential [8]. To our knowledge, large for gestational age has not been reported in the literature to be a consequence of chemotherapy.

Chemotherapy can cause maternal and/or fetal side effects [9–11]. In this article, we review the most current literature describing the effects of chemotherapy on fetal growth restriction in pregnant women with gynecological malignancies, including those of the breast, ovary, and cervix.

The investigations of fetal growth abnormalities related to chemotherapy are limited for many reasons. First, malignancy and pregnancy rarely coincide [1]. Most of the evidence in the literature comprises case reports and retrospective studies. Prospective studies are limited because of the possible effect of chemotherapy on fetuses [12]. Intending to deliver the fetus prematurely is one of the methods used to allow more aggressive chemotherapy and/or radiotherapy after labor. This causes delivered babies to risk complications of prematurity rather than of IUGR. Most cases of IUGR occur in the third trimester [3]; therefore, the exact incidence of growth abnormalities can theoretically be underestimated in such studies. The low birth weights reported in systemic reviews of certain chemotherapeutic agents may indicate prematurity with respect to the expected weight rather than pathologically decreased mass. A systemic review of 24 studies of administering platin derivatives to pregnant patients with cervical cancer revealed that the mean delivery weight of newborns was 2213 g [13]. Most other studies analyzed other consequences of chemotherapy such as congenital malformations or neonatal and/or childhood long-term complications [14]. The effect of a single chemotherapy agent can be difficult to analyze since patients can be given multiple agents [13]. The lack of precise uniform protocols of chemotherapy during pregnancy complicates the interpretation of the results. These different protocols involve different times of commencing chemotherapy, multiagent chemotherapy regimens for a single patient, use of chemotherapy alone or combined with surgery, choice of chemotherapeutics (which can be modified), dose of chemotherapy and intervals between cycles, method of administration, maternal wishes, and ethical issues [12–19]. Overlapping of chemotherapy with other factors may further complicate analysis. IUGR can also result from smoking, which is a well-known risk factor for certain malignancies such as cervical cancer [20, 21].

## 2. Effect of Chemotherapy on a Growing Fetus

The effect of the chemotherapy on a fetus may depend on the amount of the agent transferred to the fetus during pregnancy. Calsteren et al. investigated the transplacental transport of commonly used chemotherapeutics in a pregnant baboon model. The study revealed that fetal plasma concentrations of carboplatin averaged 57.5% of the maternal concentrations. Furthermore, after 3 hours of paclitaxel infusion, fetal tissue concentrations were 15% of those in the maternal tissue. As for docetaxel infusion, the fetus had 5–50% of the maternal tissue concentrations; however, the concentrations were equivalent after 26 hours. Transplacental passage of trastuzumab fell from 85% to 3% at 2 and 26 hours after trastuzumab infusion, respectively [22]. Investigations in human beings by Lanowska et al. examined the level of cisplatin in the amniotic fluid and umbilical cord blood of fetuses whose mothers underwent cisplatin monotherapy for cervical cancer during the second trimester; they found that the cisplatin concentrations in the umbilical cord and amniotic fluid were 31–65% and 13–42% of those in the maternal blood, respectively [23]. Köhler et al. assumed that a placental filtration mechanism of platinum may exist, as platinum concentrations in the umbilical cord blood and amniotic fluid were 23–65% and 11–24% of the maternal blood, respectively [24].

Chemotherapy can act directly on growing fetuses or else indirectly via the placenta [6, 25]. Chemotherapy administered after the completion of organogenesis can affect the eyes, genitalia, hematopoietic system, and central nervous system [6]. Depression of the maternal and fetal bone marrow can also cause anemia [9], which in turn can affect fetal growth [26]. Chemotherapy-induced anorexia can cause maternal nutritional deficiencies that might also contribute to growth abnormalities [2].

## 3. Role of Chemotherapy during Pregnancy

Both adjuvant and neoadjuvant chemotherapy (NACH) during pregnancy have been reported in the literature [14]. Surgery is one of the methods available for certain malignancies that do not interfere with the continuation of pregnancy, for example, in breast cancer, where chemotherapy can be administered as an adjuvant treatment [12]. Ovarian or cervical malignancies are more challenging, as total abdominal hysterectomy and bilateral salpingo-oophorectomy are the main treatments but would cause the termination of pregnancy. In such cases, NACH can be a solution for pregnant women, allowing for the treatment of the mother while preserving the life of the fetus [16, 17]. Radical surgery can be performed after delivery or following a preterm elective Caesarean section at a stage when fewer complications of premature birth can be expected [27].

Some malignancies, however, are mainly treated with chemotherapy even in nonpregnant women (mainly lymphomas). In such cases, chemotherapy can be considered for saving the lives of both the mother and fetus [8]. Odelia et al. performed a review of the literature between 1990 and 2014 on chemotherapy for lymphoma in pregnant women. They concluded that, despite a consensus regarding the safety of chemotherapy (except methotrexate) after the first trimester, optimal dosage, central nervous system therapy, timing of delivery, and approach to future pregnancies remain controversial, indicating a need for further collaborative research in this field. In most of the reviewed reports, steroids or vinblastine was suggested to be reasonable “bridging therapies” until the second trimester in patients with Hodgkin lymphoma [28].

## 4. Timing of Chemotherapy Onset

The time of chemotherapy commencement should be adjusted to increase the survival chances of the pregnant woman while decreasing the harmful effects to the fetus. Most of the observational studies analyzed the effect of chemotherapy in the second and third trimester; however, some studies analyzed the effects of chemotherapy in the first trimester. Avilés et al. investigated the risk of teratogenicity due to chemotherapy in 43 pregnant patients, 19 of whom were treated in the first trimester. However, the physical, neurological, psychological, hematological, and immune function and cytogenetics of the infant postpartum were normal. These results suggest that chemotherapy can be administered even during the first trimester of pregnancy; however, the results should be interpreted with caution because of the low number of patients in the study [15]. Moreover, García-Manero et al. reported that none of 4 pregnant patients with breast cancer who underwent chemotherapy starting in the tenth week of gestation (when organogenesis is completed) had fetuses with congenital malformations [12].

Delivering a baby, even by elective premature labor after the fetus has achieved sufficient gestational growth, can allow for additional chemotherapy and/or radiotherapy [29, 30]. After delivery, more aggressive chemotherapy can be administered to the mothers [31]. Chemotherapy in late pregnancy may not be as safe as previously assumed; a systemic review by Mir et al. suggested that platinum derivatives may cross the placenta during the final weeks of pregnancy and that close neonatal surveillance is therefore recommended in such cases [32].

## 5. Choice of Chemotherapy

Nonstandard regimens can be used if concerns arise regarding the toxic effects of the chemotherapeutic agent(s) on the fetus. Hubalek et al. reported that nonstandard carboplatin/paclitaxel chemotherapy can be used for the treatment of stage III dysgerminoma in pregnant women instead of the cisplatin, etoposide, and bleomycin (BEP) regimen, which can reportedly cause side effects (especially etoposide) [16]. Furthermore, Picone et al. reported the successful management of advanced (FIGO stage III) endometrioid carcinoma of the ovary that was diagnosed at 22 weeks of gestation; they administered only 2 courses of carboplatin before delivering the infant. After a Caesarean section was performed at 34 weeks of gestation, the therapy was continued with 7 courses of a carboplatin and paclitaxel regimen [33]. However, there is a lack of evidence on whether alternative chemotherapeutic agents can provide better treatment results or have less toxic effects on the fetus. Choosing a suitable chemotherapeutic agent may be challenging, especially when the cancer is advanced or when a relapse occurs during the first trimester. The wishes of the mother should also be considered, and the possible treatment modalities (whether chemotherapy, radiotherapy, and/or surgery) should be discussed with the patient [34]. Multidisciplinary team decision-making and an individualized plan for each case should be considered for all pregnant women with malignancies [35].

## 6. Dose and Frequency of Chemotherapy

There is insufficient evidence in the literature to ascertain whether dose modification or establishing intervals between chemotherapy cycles can improve the overall outcomes. Doi et al. successfully administered carboplatin therapy with an area under the curve of 3 plus a paclitaxel dose of 120 mg/m^2^ biweekly to an ovarian cancer patient; these overall doses were lower than those normally used [18]. However, in a study utilizing an ex vivo human placental perfusion model to predict potential fetal exposure to carboplatin during pregnancy, carboplatin doses up to an area under curve of 7.5 were not associated with significant placental transfer, fetal exposure, or toxicity to the fetus. The investigators thus suggested that it might not be necessary to empirically reduce carboplatin doses in pregnant patients [36]. Similarly, Cardonick et al. retrospectively compared a group of 10 patients who received dose-dense chemotherapy every 2 weeks to a group of 99 pregnant patients who received conventional chemotherapy, with at least 3-week intervals, for breast cancer. They found no significant differences between these groups in terms of infant birth weight and growth rate [37].

## 7. Chemotherapy for Breast Cancer during Pregnancy

There is no preferred chemotherapy regimen for breast cancer treatment during pregnancy. García-Manero et al. used the classic FAC (5-fluorouracil, doxorubicin, and cyclophosphamide) in 11 patients and found it to be safe; additionally, 4 patients were treated with taxanes with no significant complications in the patients' children postpartum [12].

In another study of 47 patients treated with the FAC regimen, only 1 child born at 29 weeks owing to maternal preeclampsia weighed less than 2000 g at birth. Six infants weighed less than 2500 g at birth, with birth weights ranging between 1389 and 2495 g at gestational ages of 29–40 weeks; 5 of these children were born following at least 38 weeks of gestation [14]. On the other hand, fetal growth abnormalities were reported by Berry et al., who described the effect of chemotherapy on pregnant women with primary or recurrent breast cancer. Chemotherapeutic agents included fluorouracil (1000 mg/m^2^), doxorubicin (50 mg/m^2^), and cyclophosphamide (500 mg/m^2^) administered every 3-4 weeks in the second and third trimesters of pregnancy. Of 24 cases, only 1 infant had a birth weight below the 10th percentile (adjusted for gestational age) [38].

Other protocols have been reported to produce different fetal growth outcomes. Sule and Ewemade reported that doxorubicin and cyclophosphamide administration for breast cancer treatment during the second trimester in a developing country did not affect birth weight [39]. Furthermore, Ring et al. retrospectively described the outcomes of 28 pregnant patients treated with chemotherapy for breast cancer at 5 hospitals in London. One patient received chemotherapy in the first trimester and had a miscarriage; 16 patients were treated with anthracycline-based chemotherapy and 12 received cyclophosphamide, methotrexate, and fluorouracil. Birth weights lower than the 10th percentile (adjusted for gestational age) were not observed among those whose data were available (17 cases); the median birth weight was 3.0 kg (range 1.4–3.5 kg) [40]. In another case study, Gottschalk et al. administered neoadjuvant trastuzumab therapy weekly starting at the 15th gestational week, in addition to 3-weekly carboplatin and docetaxel chemotherapy treatments. They found that trastuzumab caused fetal renal insufficiency. The fetus was delivered by Caesarean section at 34 weeks because of IUGR; prior to delivery, trastuzumab was discontinued after 21 weeks of gestation because of anhydramnios and nonvisualization of the fetal bladder. It is unknown whether trastuzumab itself can cause IUGR directly or indirectly by compromising renal fetal function [41].

## 8. Chemotherapy for Ovarian Cancer during Pregnancy

There are limited data in the literature regarding the use of chemotherapy for ovarian cancer during pregnancy; no single preferred chemotherapy regimen for this disease has been described for pregnant women. Some investigators used the traditional regimen of a platin derivative with paclitaxel to treat epithelial ovarian cancer. Doi et al. reported that 5 courses of carboplatin and paclitaxel chemotherapy administered during the second trimester (for the treatment of stage IC mucinous cystadenocarcinoma of the ovary diagnosed at 15 weeks of gestation) had no effect on fetal growth [18]. Furthermore, Ruiz Ramos et al. reported that a healthy infant was delivered at 38 gestational weeks after the mother received 6 cycles of neoadjuvant combined chemotherapy (carboplatin and paclitaxel) for stage III ovarian cancer between gestational weeks 16 to 36 [42]. Separately, Ferrandina et al. used only platin-derivative monotherapy for fear of fetal toxicity with multiagent chemotherapy. Their patients received 6 courses of adjuvant monotherapy with cisplatin for ovarian cancer during pregnancy following bilateral salpingo-oophorectomy, omentectomy, and appendectomy at 15 weeks of gestation; a healthy infant (3000 g) was delivered at 36 weeks of gestation [43].

Barut et al. reported administering 3 cycles of carboplatin chemotherapy for ovarian mucinous carcinoma in a patient diagnosed at 22 weeks of gestation. These courses were administered at 25, 28, and 31 weeks of gestation, and the fetus was delivered at 33 weeks weighing 2280 g [44]. A traditional protocol of BEP with a good pregnancy outcome was also reported by Karimi Zarchi et al. where a stage IIIC immature teratoma was diagnosed in a woman at 28 weeks of gestation. After administering BEP (consisting of 2 cycles of 15 mg of bleomycin, 100 mg/m^2^ per day of etoposide, and 20 mg/m^2^ per day of cisplatin) for 5 days every 3 weeks starting from the 29th week of pregnancy, a healthy 3100 g infant was delivered in gestational week 39 [45].

However, fetal growth abnormalities attributed to chemotherapy have been observed. IUGR developed in 2 of Cardonick et al.'s patients treated in the second and third trimesters with carboplatin and BEP [8]; the IUGR may have been related to the method of administration. In another patient, intraperitoneal carboplatin (area under the curve = 6; days 1 and 4) and paclitaxel (60 mg/m^2^; days 1, 8, and 15) on a 28-day cycle were administered to treat stage IIB grade III serous adenocarcinoma diagnosed at 12 weeks of gestation; this regimen was chosen after informing the patient of the lack of data and possible risks. Although 6 cycles were originally planned, the treatment lasted for 4 cycles owing to preeclampsia, thrombocytopenia, and SGA fetal weight. These results should be interpreted with caution since it is unknown whether the growth restriction was a result of preeclampsia or the direct effect of intraperitoneal chemotherapy [19].

The guidelines of the second international consensus meeting of the European Society of Gynecological Oncology (ESGO) recommend NACH in the form of carboplatin and paclitaxel for invasive epithelial ovarian cancer, as is used in nonpregnant patients. Bevacizumab is not recommended, as there is a lack of data regarding the use of this agent in pregnant women. For nonepithelial ovarian cancer, paclitaxel-carboplatin or cisplatin-vinblastin-bleomycin chemotherapy can be used instead of BEP [5].

## 9. Chemotherapy of Cervical Cancer during Pregnancy

There is no single chemotherapy protocol for the treatment of cervical cancer during pregnancy; most reports describe the use of cisplatin as monotherapy or combined with paclitaxel. Boyd et al. administered 3 courses of cisplatin NACH to a patient with stage IIB high-grade clear cell cervical carcinoma who was at 25 + 1 weeks of gestation; a healthy neonate was delivered, and no abnormalities in the child were observed during 15 months of follow-up [29]. Another group reported the successful administration of cisplatin as a monotherapy for the treatment of stage IB1 cervical cancer during the second trimester of pregnancy starting at gestational week 17. In this, 6 cycles of cisplatin (with an initial dose of 75 mg/m^2^ and a 15% reduction after the 4th cycle) were administered at 10-day intervals; no abnormalities were observed in the infant who was delivered at gestational week 32 [27]. Similarly, neoadjuvant monotherapy for stage IB1 cervical cancer in another pregnant woman (4 cycles of cisplatin 20 mg/m^2^) did not impede the normal development of the fetus [35]. This was also the case in other patients [46–48]. The feasibility of combining cisplatin with paclitaxel has also been described [49, 50]. However, despite the abovementioned reports, data remain limited and it remains premature to reach general conclusions on the safety of such regimens.

The second international consensus meeting of the ESGO recommended NACH for stage IB2 and higher cervical cancer. The currently recommended regimen includes platinum-based chemotherapy (cisplatin 75 mg/m^2^), preferably with paclitaxel (175 mg/m^2^) at 3-week intervals. Carboplatin with an area under the curve of 5-6 can be an alternative option to cisplatin as it is less toxic to the mother [5].

## 10. Conclusions

There is a lack of national guidelines and consensus regarding the optimal chemotherapy modality during pregnancy. Most of the available literature comprises case reports or retrospective studies that include a small number of patients. Prospective studies to investigate the effect of chemotherapy on intrauterine fetal growth are lacking. Fetal growth abnormalities are recognized sequelae of chemotherapy, and the possibility of fetal growth abnormalities as well as the other side effects of different chemotherapeutic agents should be discussed with the patients. The final decision regarding treatment should consider the type of malignancy and its stage, the use of surgery and/or radiotherapy, the stage of pregnancy, the probability of side effects during treatment, and the patient's own wishes. Centralization of treatment for these patients may help to develop a plan for a prospective study to assess all the associated factors and consequences of chemotherapy during pregnancy, particularly fetal growth abnormalities. Moreover, ethical concerns cannot be ignored. Each patient should ultimately be managed individually with the guidance of a multidisciplinary team.
